# Advanced Te_x_S_y_-C Nanocomposites for High-Performance Lithium Ion Batteries

**DOI:** 10.3389/fchem.2021.687392

**Published:** 2021-05-25

**Authors:** Guolong Lu, Chunnuan Ye, Wenyan Li, Xuedong He, Guang Chen, Jun Li, Huile Jin, Shun Wang, Jichang Wang

**Affiliations:** ^1^ Nano-materials & Chemistry Key Laboratory, Institute of New Materials and Industrial Technologies, Wenzhou University, Wenzhou, China; ^2^ Department of Chemistry and Biochemistry, University of Windsor, Windsor, ON, Canada

**Keywords:** sulfur telluride materials, electrochemical synthesis, composite materials, carbon nanotubes, lithium ion batteries

## Abstract

This study is dedicated to expand the family of lithium-tellurium sulfide batteries, which have been recognized as a promising choice for future energy storage systems. Herein, a novel electrochemical method has been applied to engineer micro-nano Te_x_S_y_ material, and it is found that Te_x_S_y_ phases combined with multi-walled carbon nanotubes endow the as-constructed lithium-ion batteries excellent cycling stability and high rate performance. In the process of material synthesis, the sulfur was successfully embedded into the tellurium matrix, which improved the overall capacity performance. Te_x_S_y_ was characterized and verified as a micro-nano-structured material with less Te and more S. Compared with the original pure Te particles, the capacity is greatly improved, and the volume expansion change is effectively inhibited. After the assembly of Li-Te_x_S_y_ battery, the stable electrical contact and rapid transport capacity of lithium ions, as well as significant electrochemical performance are verified.

## Introduction

Lithium-tellurium (Li-Te) batteries have attracted increasing attention owing to their high theoretical volume capacity (Liu et al., 2014; Ding et al., 2015; Li et al., 2017; Li G. et al., 2018; Yin et al., 2018; Wenjie Han et al., 2021), excellent electronic conductivity (He et al., 2017), and relieved shuttle effects compared to Li-sulfur, Li-selenium batteries (Yang et al., 2013; Eftekhari, 2017; Li Y. et al., 2018; Fan et al., 2019; Wang et al., 2020; Yu et al., 2020; Dai et al., 2021; Sun et al., 2021; Xiao et al., 2021). However, the huge volume expansion of Te severely deteriorates its practical applications towards the newly emerged battery systems. Therefore, how to alleviate or eliminate the volume variation is of great importance to fulfill the promising properties of Te. Since our first introduction of Li-Te_x_S_y_ battery (Li J. et al., 2019), it seems there is a hope to light a new path to conquer the volume expansion challenge by the incorporation of sulfur elements inside tellurium lattice. Although our prepared Li-Te_x_S_y_ cathode materials were not perfectly composed of pure Te_x_S_y_ phase, it has been demonstrated such Te_x_S_y_ phase is surprisingly stable in terms of *in situ* TEM observation, which can be survived during the repetitive cycling without obvious volume variation.

Many related works have tried to map the phase diagram of Te_x_S_y_, such as Te_0.92_S_0.08_, Te_0.04_S_0.96_, Te-n-S (where n represents the mass ratio) (Xu et al., 2018; Li et al., 2019a; Li et al., 2019b; Ge and Yin, 2019; Lee et al., 2019; Zhang et al., 2020). Sulfur incorporation leads to lattice distortion and d-spacing enlargement of Te phase, rendering the composited Te_x_S_y_ with a fast transport of ions and electrons, as well as excellent structural stability during lithiation/delithiation processes (Chen et al., 2020). Together with the superior electronic conductivity and enhanced reaction kinetics derived from Te, Li-Te_x_S_y_ batteries exhibit extraordinary energy storage performance and foreseeable bright future for next-generation battery systems.

In this work, we have attempted to design new types of Te_x_S_y_ phases and fill some blank in Te_x_S_y_ phase diagram by applying different kinds of sulfur sources during the nonlinear electrochemical synthesis of Te_x_S_y_ (Li et al., 2019a). The experimental results suggested that different sulfur sources give rise to distinguished lattice distortions of Te, and thus different types of Te_x_S_y_ phases, among which, Na_2_S-derived Te_x_S_y_ ball milled with multi-walled carbon nanotubes endows Li-Te_x_S_y_ batteries profound volumetric capacity performance and high cycling stability.

## Materials and Methods

### Synthesis of Te_x_S_y_ Micro-nano Materials

Sodium sulfide (Na_2_S·9H_2_O), tellurium ingot (Te) and sodium hydroxide (NaOH) were all purchased from Aladdin. The sintered Te rod, platinum wire and calomel electrode (Hg/HgCl_2_) was used as the working, counter and reference electrode, respectively. The three-electrode system was employed in an equilateral triangle manner with a distance of 1.8 cm. Before experiments, the working and counter electrodes were cleaned with ultrasonic cleaner (Branson 1510, United States) for 10 min, and then rinsed with distilled water. The temperature of reaction cell was maintained at 25.0°C. The electrochemical synthesis experiments were carried out at the CHI 660e Electrochemical Workstation (Shanghai Chenhua). A typical solution preparation is to dissolve 0.5 mol L^−1^ NaOH first, and then add 0.5 mol L^−1^ Na_2_S·9H_2_O (other sulfur sources with different concentrations were specified) to the solution to get a clear solution. The voltage window of 0–1.5 V was set by cyclic voltammetry (CV) with a scan rate of 0.1 mV s^−1^, and the electrochemical reaction was carried out by 3 CV cycles. The black solid products were finally collected, cleaned and centrifuged, which was later identified as Te_x_S_y_ micro-nano materials.

### Synthesis of Te_x_S_y_-C Nanocomposites

The above as-prepared Te_x_S_y_ materials were mixed with certain mass ratio of multi-walled carbon nanotubes (purchased from XFNANO, 50 µm in length, 8–15 nm in diameter, purity>95%) by using a ball milling machine (QM-3C, Nanjing University). After fully mixing for 20 h, the composites of Te_x_S_y_-multi-walled carbon nanotubes (Te_x_S_y_-C) were obtained.

### Characterization

Scanning electron microscopy (SEM) was performed on a Nova Nanosem 200 system with an acceleration voltage of 15 kV. Transmission electron microscopy (TEM) and high resolution transmission electron microscopy (HRTEM) were conducted on JEM-2100F. Energy dispersive X-ray energy spectrum (EDX) and TEM measurements were performed simultaneously. Raman spectroscopy (INVIA, Renishaw, United Kingdom) was carried out at an ambient temperature with a 514 nm laser excitation. X-ray photoelectron spectroscopy (XPS) was performed in the spectrometer from Kratos axis Ultradld, using Mono Al Ka radiation power of 120 W (8 mA, 15 kV). X-ray diffraction (XRD) was tested by using a Cu-Ka radiation (A = 0.15406 nm) on the Bruker D8 Advanced Diffractometer with a data acquisition range of 10°–80° and sweep rate of 0.02° s^−1^. Thermogravimetric analysis (TGA) was performed on Perkin-Elmer PRIS1 TGA/Clarus SQ 8T at a heating rate of 5°C min^−1^.

### Electrochemical Test

The electrochemical properties of Te_x_S_y_-C nanocomposites were studied by using the 2025 coin battery on the Neware-battery testing system. The working electrode was prepared by pasted a mixture of 70 wt% Te_x_S_y_-C nanocomposites, 15 wt% acetylene black and 15 wt% polyvinylidene fluoride (PVDF) on the aluminum foil. The mass loading of the active material on the electrode was 1–2 mg cm^−2^, and the lithium metal wafer was used as the counter electrode. The electrolyte was containing 1 mol L^−1^ lithium bis(trifluoromethane)sulfonimide (LiTFSI) electrolyte, 2% LiNO_3_, and 1,3-dioxolane (DOL) and 1,2-dimethoxyethane (DME) (volume ratio = 1:1). The battery was assembled in a glove box filled with pure argon gas.

## Results and Discussion

**FIGURE 1:**
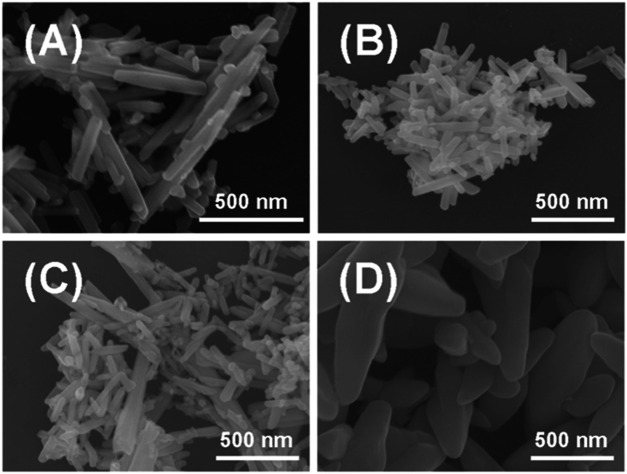
SEM image of solid products prepared by electrochemical cyclic voltammetry with different types of precursor S sources and the same concentration of 0.5 mol L^−1^: **(A)** Te@C_3_H_6_NNaS_2_, **(B)** Te@CH_4_N_2_S, **(C)** Te@NaHS, **(D)** Te@Na_2_S.

In this work, we have tried various sulfur sources to fabricate different types of Te_x_S_y_ phases via a nonlinear electrochemical approach. By changing the actively reducing sulfur species such as sodium dimethyldithiocarbamate (C_3_H_6_NNaS_2_) and thiourea (CH_4_N_2_S), sodium hydrogen sulfide (NaHS) and sodium sulfide (Na_2_S) for the production of Te_x_S_y_ phases, distinctive micro-nano structured Te_x_S_y_ materials were engineered in Figure 1 via the control of nonlinear electrochemical dynamics in Supplementary Figure S2. The scanning electron microscope (SEM) images indicated that the presence of Na_2_S could lead to a distinguished morphology (flakes) compared to that of other products (rods). More importantly, Raman spectra in Supplementary Figure S3 revealed that the sulfur content was maximized in Te@Na_2_S, denoted as Te_x_S_y_ phases prepared by Na_2_S. The optimal concentration of Na_2_S for the construction of nano-flaked Te_x_S_y_ phases was determined as 0.5 mol L^−1^. As the concentration of Na_2_S was increased, the nano-flaked Te_x_S_y_ phases were broken into randomly downsized nano-particles, as shown in Supplementary Figure S4. Surprisingly, the increasing concentration of Na_2_S also led to an overwhelmed Raman peak intensity of sulfur than tellurium in Supplementary Figure S5.

**FIGURE 2:**
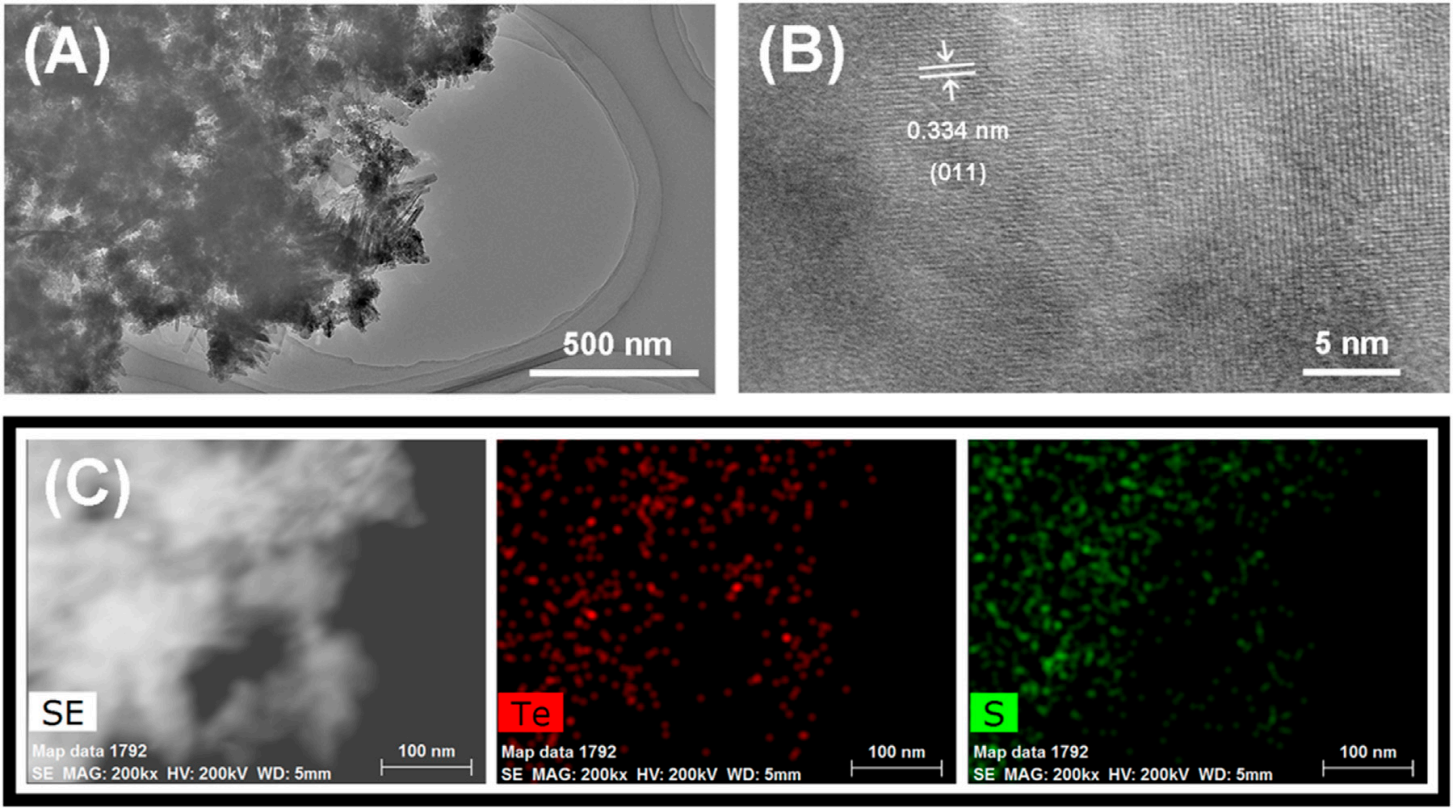
Characterization of Te_x_S_y_: **(A)** TEM, **(B)** HRTEM, **(C)** EDS.

Transmission electron microscopy (TEM) characterization of Te_x_S_y_ material was obtained when the concentration of Na_2_S was set to 2.0 mol L^−1^. It can be found that the downsized nanoparticles have a very poor crystallinity from Figures 2A,B, in which a typical lattice parameter is emerged in a typically selected area, with a d-spacing of 0.334 nm representing Te (011) plane. The observed lattice spacing agrees with the hexagonal element Te phase. Therefore, the as-obtained materials are not composed of pure Te_x_S_y_ phases. Another evidence is from XRD analysis in Supplementary Figure S5B, where the XRD peaks are broadened as the concentration of Na_2_S is increased, indicating the incorporation of sulfur in Te crystalline causes the poor crystallinity. Furthermore, Te_x_S_y_ phases are dominated in the as-prepared materials, strongly supported by the homogeneous distribution of Te and S elements in Figure 2C.

**FIGURE 3:**
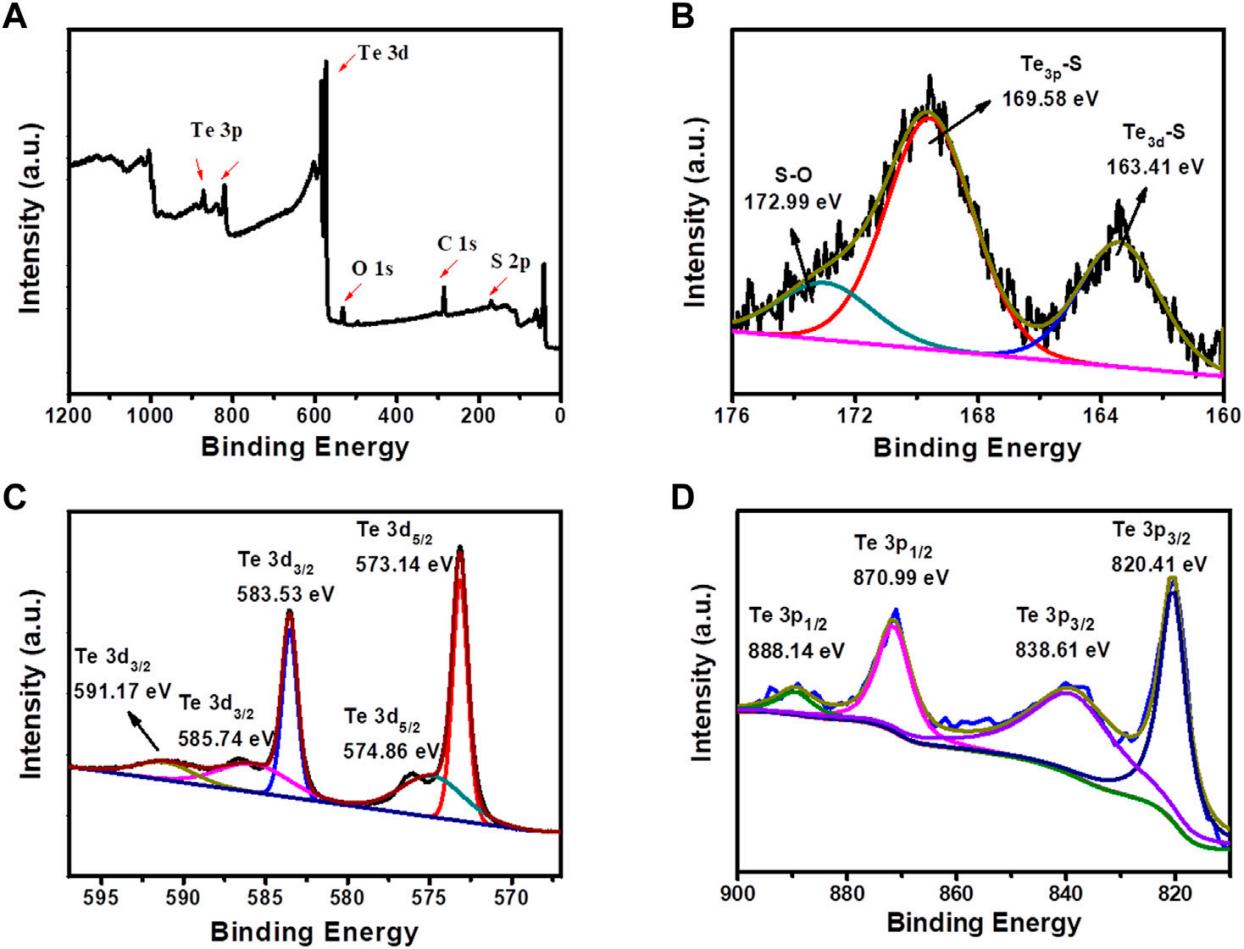
XPS spectra of Te_x_S_y_ products prepared at Na_2_S concentration of 2.0 mol L^−1^: **(A)** Survey spectrum, **(B)** S 2p, **(C)** Te 3d, **(D)** Te 3p.

In order to further study the composition of Te_x_S_y_ component and the formation mechanism of Te-S bond, the product synthesized by 2.0 mol L^−1^ Na_2_S solution was selected for XPS characterization (Figure 3). Figure 3A presented a XPS survey image of Te_x_S_y_ components possessing the main elements of Te and S. Figure 3B displayed three types of sulfur bonding, where 163.41, 169.58 and 172.99 eV are the electron binding energies of Te_3d_-S bond, Te_3p_-S bond and S-O bond respectively. It can be seen from Figures 3C,D that there were multiple types of Te oxidized states. 573.14 and 574.86 eV are the electron binding energies of Te 3d_5/2_ bond, and 583.53, 585.74 and 591.17 eV are the electron binding energies of Te 3d_3/2_ bond, demonstrating that Te element in Te_x_S_y_ component exists in the form of Te^4+^ and Te^6+^. Therefore, it can be speculated that the formation of Te-S bond is derived from the electrochemical oxidation of Te on the main electrode to form TeO_4_^2−^ and TeO_3_^2−^, which are then chemically reduced by different organic or inorganic sulfides in this study to form Te_x_S_y_. The overall reaction mechanism is followed by the electrochemical-chemical (EC) reaction pathway, similar as the first two steps of our previous study (Li J. et al., 2019). The distinguished reducibility of organic and inorganic sulfides enabled the self-assembly of Te_x_S_y_ with different nano-micro morphologies and chemical compositions, rendering Te_x_S_y_ with varied physicochemical properties for seeking the promising electrochemical performance.

However, compared to our previous study (Li J. et al., 2019), the as-prepared Te_x_S_y_ phases without any confinements from carbon hosts were failed to contribute a promising electrochemical performance towards lithium ion batteries. As shown in Supplementary Figure S6, high charge transfer resistance, poor cycling stability and rate performance seems a total failure. Therefore, in this work we applied multi-walled carbon nanotubes (MWCNTs) as a carbon host to confine the Te_x_S_y_ phases. Astonishingly, the ball milling of Te_x_S_y_ phases with MWCNTs rendered the Te_x_S_y_(Na_2_S)/MWCNT with a strange thermal degradation feature, that is, Te_x_S_y_ phases actually reacted with MWCNTs, and resulted in a two-stage thermal degradation of Te_x_S_y_ in Supplementary Figure S8B. In comparison with Te_x_S_y_, the Te_x_S_y_-C possessed a distinctive thermal degradation kinetics, where the less mass ratio of Te_x_S_y_:C such as 5:5 and 3:7 could lead to the lower decomposition temperatures (∼630°C as shown in Supplementary Table S1) than that of pure Te_x_S_y_ and the high mass ratio of Te_x_S_y_:C = 7:3 (∼700°C), indicating the rearrangement of Te_x_S_y_ phases was occurred in the presence of appropriate amounts of MWCNTs.

**FIGURE 4:**
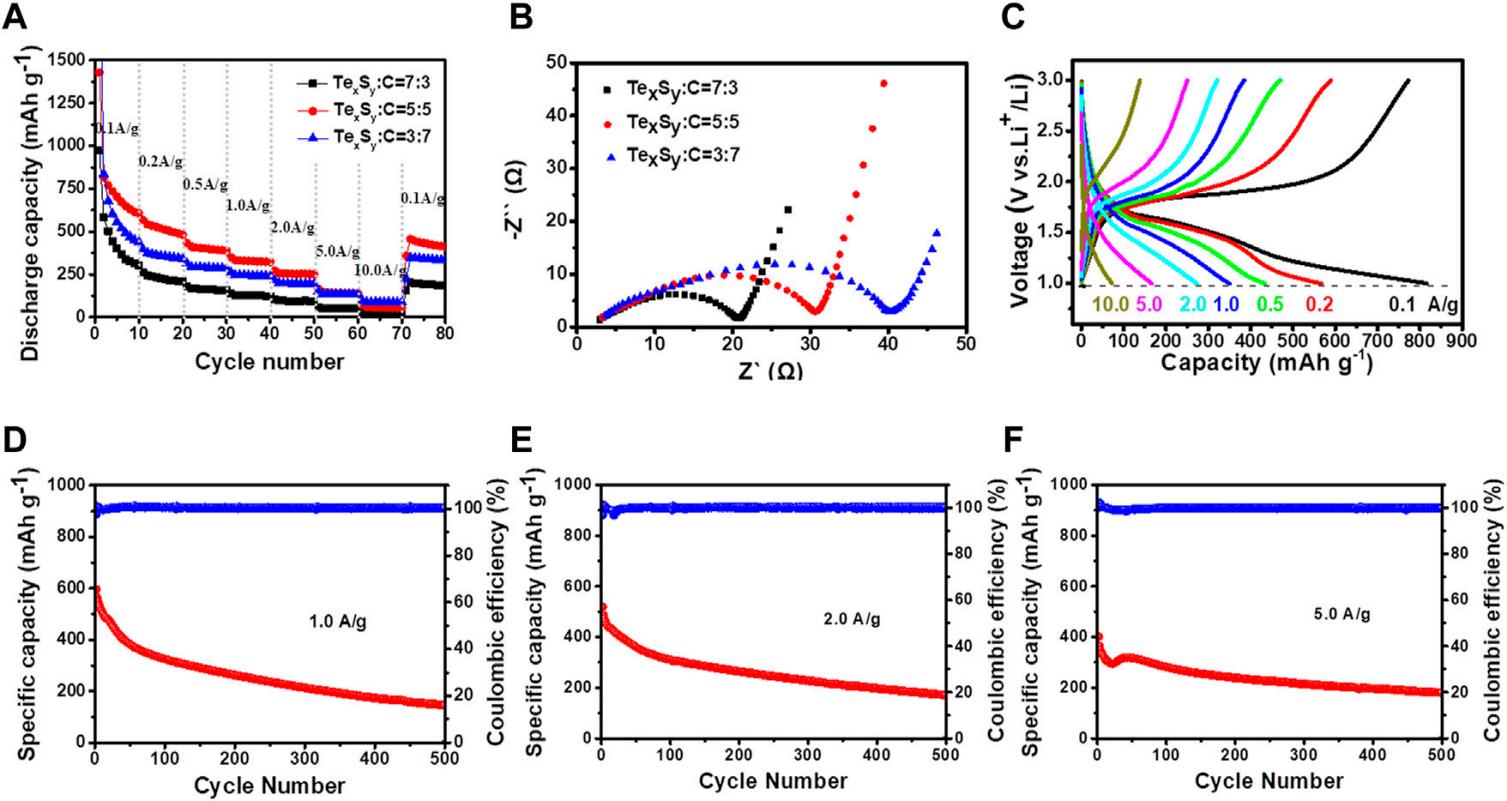
The electrochemical performance of Te_x_S_y_(Na_2_S)/MWCNT composites: **(A)** rate performance, **(B)** EIS measurements, **(C)** charging/discharging curves at varied current densities towards the mass ratio of 5:5 Te_x_S_y_(Na_2_S)/MWCNT composites, cycling stability and Coulomb efficiency of 5:5 Te_x_S_y_(Na_2_S)/MWCNT composites at the current density of **(D)** 1.0 A g^−1^, **(E)** 2.0 A g^−1^, **(F)** 5.0 A g^−1^.

Afterwards, the Te_x_S_y_(Na_2_S)/MWCNT composited materials with different mixing ratios were further evaluated by battery performance test, as shown in Figure 4. Figure 4A showed a diagram of the rate performance of Te_x_S_y_(Na_2_S)/MWCNT composites with different mixing ratios. It is worth noting that the materials with composite ratio of 5:5 have relatively better rate performance than the other two materials. Electrochemical impedance spectroscopy (EIS) of Te_x_S_y_(Na_2_S)/MWCNT composited batteries with different mixing ratios were also tested in Figure 4B. As it can be seen, the increased amount of multi-walled carbon nanotubes would worsen the charge transfer, suggesting an integrated interfacial effects between Te_x_S_y_ and MWCNTs. In addition, Figure 4C showed the charge-discharge curves of lithium ion battery with a mixture ratio of 5:5 at different current densities. The first charge-discharge curves suggest a relatively high initial Coulombic efficiency. Figures 4D–F presented that the compound ratio of 5:5 Te_x_S_y_(Na_2_S)/MWCNT composited battery exhibits a promising cycling behavior at 1.0, 2.0, 5.0 A g^−1^, in which a larger current density would lead to a better cycling stability, indicating a potential fast-charging application in rechargeable batteries. When the current density was set to 5.0 A g^−1^, the first specific capacity was obtained as 406.56 mAh g^−1^, and capacity retention remained as 45.03% after 500 cycles. While the current density was set less than 5.0 A g^−1^, the rate performance behaved much worse than that at 5.0 A g^−1^, demonstrating Te_x_S_y_(Na_2_S)/MWCNT composite material is more suitable for high rate performance in lithium ion batteries. The as-prepared lithium-tellurium sulfide battery may potentially tackle the cons of low rate performance in lithium-sulfur battery, and high volume expansion and low capacity performance in lithium-tellurium battery.

## Conclusion

In summary, we designed a promising electrochemical method to control the synthesis of Te_x_S_y_ micro-nano structured composites, verified the formation mechanism and qualitatively evaluated the influence of chemical composition on the battery performance. The morphology and composition ratio of Te_x_S_y_(Na_2_S)/MWCNT were controlled by the types of sulfur sources, concentration and synthetic voltage. In addition, MWCNTs as an ideal carbon host were used for the confinement of the dissolution of tellurium and sulfur, which significantly improved the electrochemical performance of the Te_x_S_y_(Na_2_S)/MWCNT composited battery. The nonlinear electrochemical synthetic method and ball milling aftertreatments provide a new way for the sustainable development of high-performance Li battery manufacturing.

## Data Availability

The original contributions presented in the study are included in the article/Supplementary Material, further inquiries can be directed to the corresponding authors.
