# Supplementary material for: Advanced TexSy-C Nanocomposites for High-Performance Lithium Ion Batteries
Source: Front Chem. 2021 May 25;9:687392. doi: 10.3389/fchem.2021.687392 (PMC8186662; doi:10.3389/fchem.2021.687392)
Supplement: Supplementary file 1 [file DataSheet1.docx]

Supplementary Material

## Supplementary Figures

**
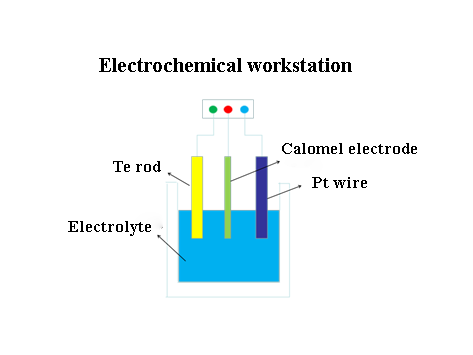
**

**Supplementary Figure 1.** Diagram of sample synthesis process.


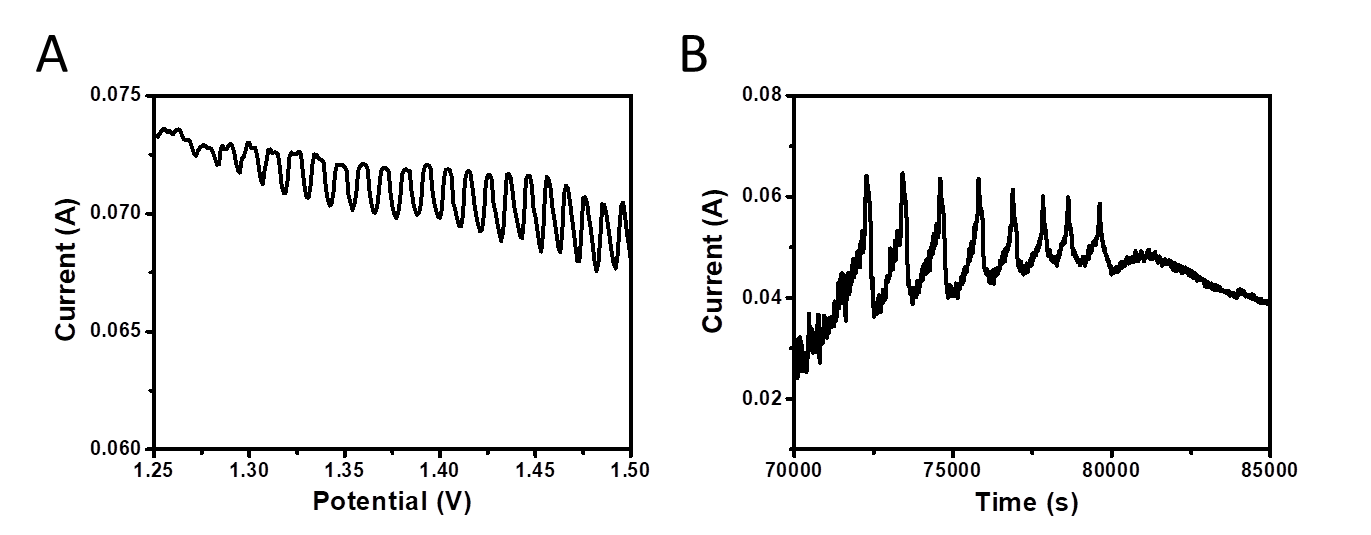


**Supplementary Figure 2.** (A) Nonlinear electrochemical CV curve in 0.5 mol L^-1^ Na_2_S solution; (B) Nonlinear electrochemical i-t curve in 0.5 mol L^-1^ Na_2_S solution at 1.1 V.


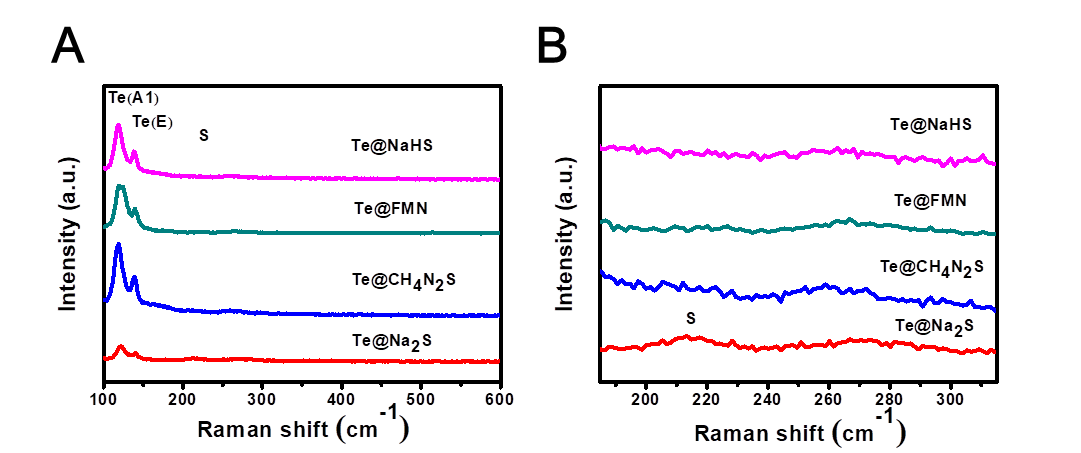


**Supplementary Figure 3.** (A) and (B) are Raman spectra of solid products prepared by electrochemical cyclic voltammetry with different types of precursor S sources, and the same concentration of 0.5 mol L^-1^.


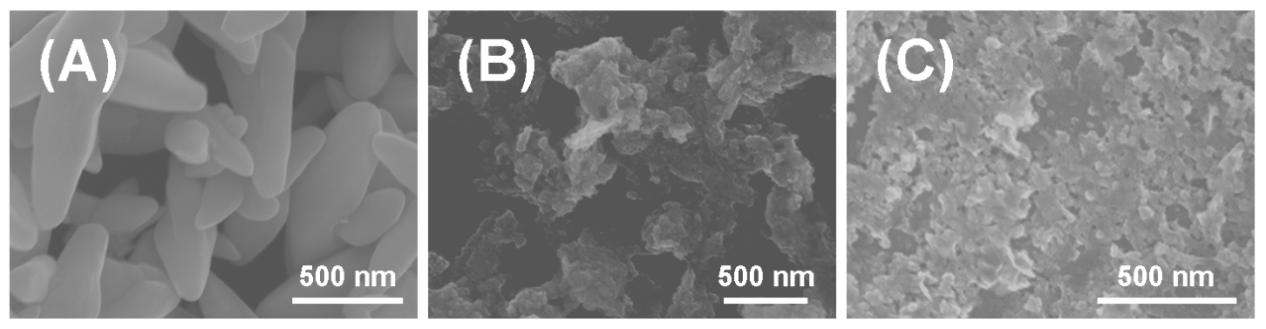


**Supplementary Figure 4.** SEM images of Te_x_S_y_ products prepared by electrochemical cyclic voltammetry at different concentrations of Na_2_S solution concentrations: (A) 0.5 mol L^-1^, (B) 1.0 mol L^-1^, and (C) 2.0 mol L^-1^.


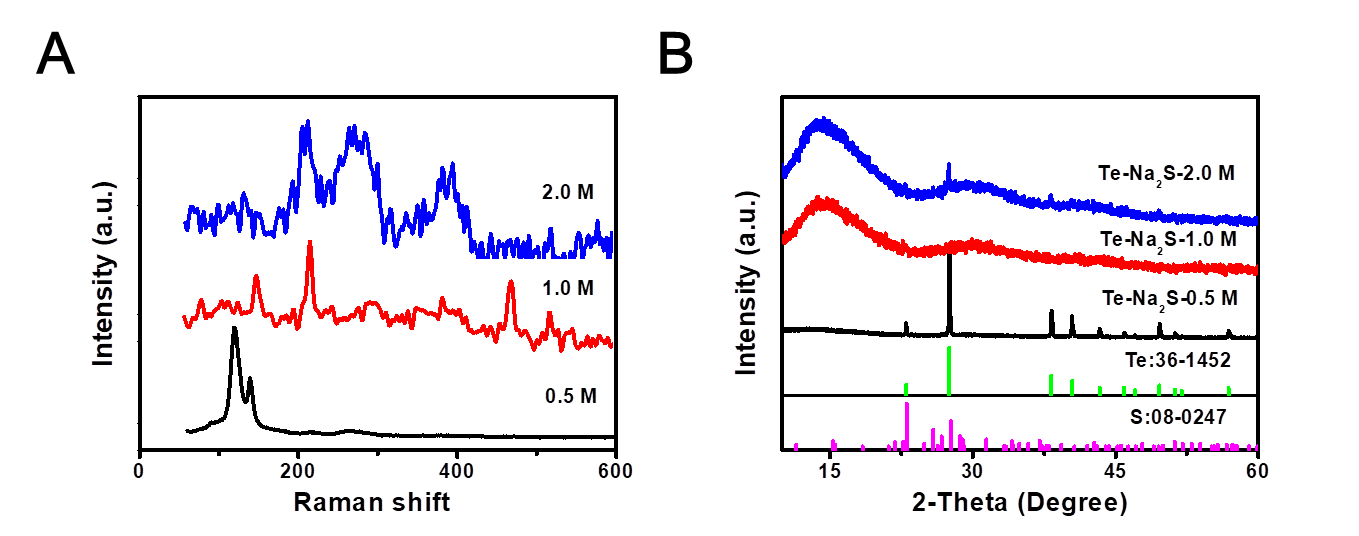


**Supplementary Figure 5.**  (A) and (B) is Raman diagram and XRD patterns of Te_x_S_y_ prepared at different concentrations of Na_2_S solutions.


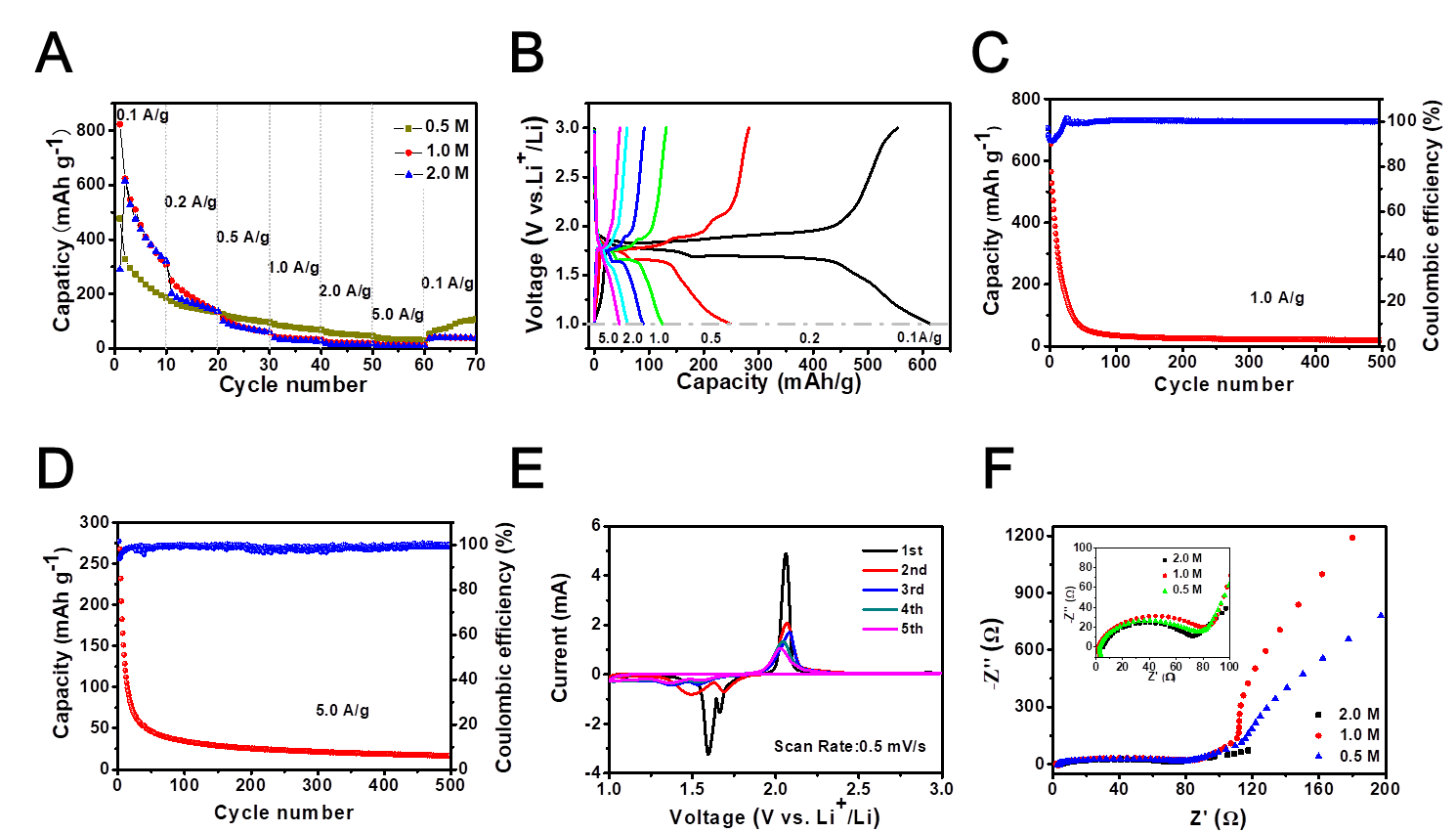


**Supplementary Figure 6.** Electrochemical performance of Te_x_S_y_: (A) Rate perfoamance, (B) Discharge and charge curves at the different rate of the Te_x_S_y_ prepared in 2.0 mol L^-1^ Na_2_S Solution, (C) Cyclic stability and Coulombic efficiency of Te_x_S_y_ prepared in 2.0 mol L^-1^ Na_2_S solution after 500 cycles at 1.0 A g^-1^, (D) Cyclic stability and Coulombic efficiency of Te_x_S_y_ prepared in 2.0 mol L^-1^ Na_2_S solution after 500 cycles at 5.0 A g^-1^, (E) Cyclic Voltammograms of Te_x_S_y_ prepared at different concentrations of Na_2_S solution at a scan rate of 0.5 mV s^-1^, (F) EIS profiles of Te_x_S_y_ prepared at different concentrations of Na_2_S solution at an open circuit potential.


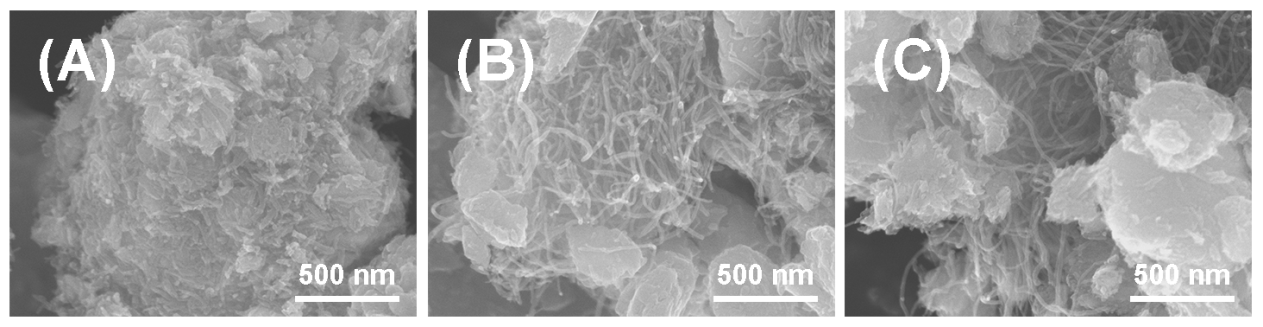


**Supplementary Figure 7.** SEM characterization of Te_x_S_y_(Na_2_S)/MWCNT product the mass ratios of Te_x_S_y_ composite to MWCNT are (A) 7:3, (B) 5:5, and (C) 3:7, respectively.


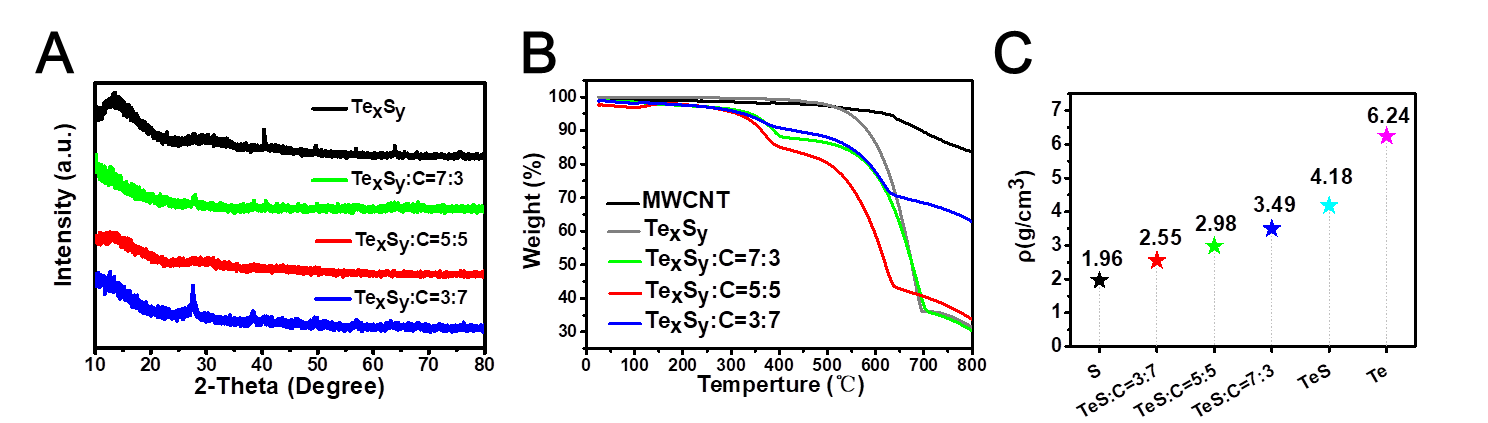


**Supplementary Figure 8.** (A) XRD characterization of Te_x_S_y_(Na_2_S)/MWCNT composites with different mixing ratios; (B) TG analysis curves of Te_x_S_y_(Na_2_S)/MWCNT composites with different mixing ratios; (C) True density of S, Te_x_S_y_(Na_2_S)/MWCNT, Te_x_S_y_ and Te materials with different mixing ratios.

**Supplementary Table 1.** Thermal weightlessness platform temperature and weightlessness percentage of Te_x_S_y_-C composites with different mixing ratios.

| **Materials** | **T_1_** | **Weight**  **loss** | **T_2_** | **Weight**  **loss** |
| --- | --- | --- | --- | --- |
| Te_x_S_y_:C=7:3 | 390℃ | 7.37% | 700℃ | 49.48% |
| Te_x_S_y_:C=5:5 | 390℃ | 8.15% | 635℃ | 36.73% |
| Te_x_S_y_:C=3:7 | 390℃ | 6.22% | 630℃ | 22.18% |
